# Effects of Personality Types on the Performance of Educational Teams

**DOI:** 10.3390/bs15030312

**Published:** 2025-03-05

**Authors:** Juan Manuel Criado, Gema Gutiérrez, Emilio L. Cano, Javier Garzás, María Teresa González de Lena, Javier M. Moguerza, Juan José Fernández Muñoz

**Affiliations:** 1 Department of Computer Science and Statistic, Rey Juan Carlos University, 28933 Mostoles, Madrid, Spain; gema.gutierrez@urjc.es (G.G.); emilio.lopez@urjc.es (E.L.C.); javier.garzas@urjc.es (J.G.); mariateresa.gonzalezdelena@urjc.es (M.T.G.d.L.); javier.moguerza@urjc.es (J.M.M.); 2 Department of Psychology, Rey Juan Carlos University, 28933 Mostoles, Madrid, Spain; juanjose.fernandez@urjc.es

**Keywords:** personality types, team performance, innovation, self-managing work team, Enneagram

## Abstract

The objective of this study is to explore how various personality types correlate with enhanced work performance. The Enneagram type of the participants in the experiment was established by using the simplest version of the Riso–Hudson test. A two-way ANOVA was performed under the principles of the Design of Experiments, which allowed the identification of main effects and interactions in the response, i.e., the marks of the university teams. We found that the interactions between certain Enneagram types seem to increase the average performance marks as a primary effect. Conversely, when certain Enneagram types coincided within a team, the marks significantly decreased, posing a risk to project success. According to our results, the Enneagram framework may be used as a preliminary stage for identifying potential team members for future projects.

## 1. Introduction

The efficiency of work teams is a central topic in both academic and professional settings, as organizations increasingly rely on effective collaboration among team members to achieve their goals. Factors such as communication, decision-making, and conflict resolution are essential; however, one of the most decisive elements in team performance is the personality types of its members, as group dynamics can significantly influence project success and collective learning (Tuckman, 1965). Previous studies (Conard, 2006; Guzzo & Shea, 1992) suggest that understanding personality interactions within a team can be key to improving cohesion and performance. In this context, the present study aims to explore how personality models can be effectively applied in academic settings to create high-performing teams.

Through an analysis of existing theories and an assessment of the impact of personality traits on academic achievement, this paper offers an innovative perspective on fostering collaboration and improving outcomes in diverse teams (Snow & Walton, 2018). Thus, the dependent variable in this study is the final mark obtained by each team. The study investigates how different combinations of personality types may affect the teams’ performance and their final grades.

Regarding the independent variables, these will be the so-called enneatypes (personality types): reformer, helper, achiever, individualist, investigator, loyalist, enthusiast, challenger, and peacemaker. These enneatypes and how they are determined will be explained in detail later. The relationship between personality types and the response variable is crucial for understanding the role of emotional and motivational factors in improving team performance.

The objective of this study is to explore how various personality types correlate with enhanced work performance. The hypothesis is that there is a significant relationship between the personality types of team members and the final outcome of their joint work. This document begins by reviewing theoretical and empirical research concerned with personality types and traits. The method section provides an overview of the methodological approach with the characteristics of the sample used, the procedures followed, and the instruments applied to the participants. In particular, for the identification of main effects and interactions in the response, an Analysis of Variance (ANOVA) was performed. The results section includes the findings of the data analysis from the questionnaire and discusses the experimental findings. Finally, a discussion offers the main conclusions, limitations, and further research.

### Related Works

The Five Factor Model, commonly referred to as the Big Five, represents a significant milestone in the understanding of personality types (Eysenck, 1959; Eysenck & Eysenck, 1975; Tupes & Christal, 1992; Carlyn, 1977; Cattell et al., 1970; Costa & McCrae, 1992). This model categorizes personality traits into five distinct dimensions: emotional stability, extraversion, openness, agreeableness, and conscientiousness (Eysenck, 1998). Each dimension captures a broad spectrum of human personality and behavior, providing a comprehensive framework for studying individual differences.

Emotional stability, also known as neuroticism, reflects an individual’s tendency to experience negative emotions, such as anxiety, depression, and irritability. Extraversion encompasses traits related to sociability, assertiveness, and positive emotionality. Individuals high in extraversion seek social interaction and environmental stimulation to a greater extent than those low in extraversion. Openness to experience signifies a person’s receptiveness to new ideas, creativity, and intellectual curiosity, while agreeableness refers to qualities such as kindness, empathy, and cooperation in interpersonal relationships. Conscientiousness reflects self-discipline, organization, and goal-directed behavior, indicating a person’s ability to plan, manage tasks, and adhere to rules and obligations (Eysenck, 1959, 1998; Eysenck & Eysenck, 1975). It should be clarified that individuals high in extraversion seek social interaction and environmental stimulation to a greater extent than those low in extraversion.

The Big Five has been applied in various fields of study. On the one hand, Parra et al. (2022) analyzed how the five major personality traits (neuroticism, extraversion, openness to experience, agreeableness, and conscientiousness) affect work exhaustion in the context of remote work. Specifically, the authors examined how different combinations of these personality traits may influence the experience of exhaustion among employees working remotely. On the other hand, Espinoza et al. (2023) investigated how personality types related to the Big Five influence individuals’ conflict management styles. This study provides a deeper understanding of how different aspects of personality may affect how people handle conflicts in their interpersonal and work relationships.

In the engineering environment, Rodrigues and Rebelo (2013) conducted a study to assess the incremental validity of proactive personality over the Big Five in predicting job performance of software engineers in an innovative context. They examined whether proactive personality provided additional information beyond what can be predicted using the Big Five personality traits in the field of software engineering.

The Riso–Hudson Enneagram Type Indicator (RHETI) is a tool used in the field of computer engineering to assess personality types through nine basic profiles, or enneatypes. It provides valuable insights into individual strengths and weaknesses within the context of software development teams. The RHETI offers two versions: one comprising 144 paired statements and an abbreviated version featuring only two questions. These questions are validated by various authors within the software engineering community. Each question presents three options, aligning with one of nine personality types: reformer, helper, achiever, individualist, investigator, loyalist, enthusiast, challenger, and peacemaker. While research on the Enneagram’s application specifically in software engineering teams is limited, some studies have explored its utility in similar technical environments (Daniels & Price, 2000). This, combined with its license-free nature, makes the Enneagram a compelling choice for personality assessment in computer engineering contexts.

In educational or coaching contexts, where self-awareness is crucial, the Enneagram can enhance communication and group dynamics (McCloskey, 2013). Although the Enneagram has had more limited application in academic settings compared to models like the Big Five, and the finding may be affected by its internal validity, its ability to complement other assessments and its versatility make it a relevant and effective tool for in-depth personality exploration. Our choice is based on several key reasons: (i) The Enneagram not only provides an understanding of personality types but also incorporates motivational and emotional aspects. (ii) Despite its limited application in academic research so far, the Enneagram has gained popularity in various practical settings, including personal development, therapy, and coaching, suggesting untapped potential for its application in education.

Personality models, particularly the RHETI model, have been explored in the literature to enhance teamwork, as demonstrated by Bonilla et al. (2008). Regarding the educational context, Bonilla, Lord, and Perry proposed a web-based application for assessing students’ personality profiles using the DISC assessment method. Agarwal and Kwuimy (2021) conducted a study on personality and performance in a semi-virtual learning environment, focusing on teams composed of individuals with varying levels of traits, such as extraversion, openness to experience, conscientiousness, agreeableness, and neuroticism. The study suggests that diversity in these traits can significantly influence team performance, with certain traits showing a positive correlation with effective performance in environments that combine both in-person and virtual components. Other researchers, including Komarraju et al. (2011), investigated the impact of personality models on academic performance. Their study found that certain personality traits, such as conscientiousness and openness to experience, are positively correlated with academic success. The research also highlighted that different learning styles mediate the relationship between personality traits and academic performance, suggesting that understanding these dynamics can help improve educational outcomes.

From an organizational psychology perspective, the functional theory of group decision-making, team composition theory, and consensus decision-making theory highlight key factors for improving team performance. The functional theory emphasizes task-oriented roles and communication for effective decision-making (Gouran & Hirokawa, 1996), while team composition theory suggests that a diverse mix of skills and experiences within a team enhances its performance (Belbin, 2010; Gutiérrez et al., 2019; Henry & Stevens, 1999). Additionally, consensus decision-making fosters cohesion and commitment by ensuring that all team members support the final decision (McGrath, 1984). These theories suggest that team success depends on effective role distribution, diversity, and collective agreement in decision-making.

Building on these theories, the integration of the Big Five personality traits and the Enneagram types provides a deeper understanding of how individual differences influence team dynamics. The Big Five traits—such as extroversion, agreeableness, and neuroticism—can affect how team members interact, contribute to decision-making, and manage group processes. The Enneagram, with its focus on core motivations and behaviors, further enriches this understanding by offering insights into how members with different personality types may approach team tasks and decision-making (McCrae & Costa, 1997; Riso & Hudson, 1999b).

Within this framework, the following hypotheses were raised:

**H_1_.**  *The combination of certain enneatypes in a team will significantly improve the final marks.*

**H_2_.**  *The combination of certain enneatypes in a team will significantly decrease the final marks.*

## 2. Methodology

### 2.1. Sample

The sample includes 165 students from four topics taught in the Computer Engineering Degree at a Spanish university (164 valid responses). In total, 79.39% were male, 15.76% were female, and 4.85% did not respond. The degree consists of four levels, and all of these subjects are taught in the third level. The name of the topics are Software Processes (SPs), with 53 participants (32.12%), distributed in 9 teams; Software Quality (SQ), with 61 participants (36.98%, one of them had missing data), distributed in 10 teams; Software Architecture Design (SAD), with 32 participants (19.39%), distributed in 6 teams; and Project Management (PM), with 19 participants (11.51%), distributed in 4 teams. Chronologically, the Software Quality course is taught first, followed by the Software Architecture and Software Processes courses, which are taught in parallel in the next term. In the following academic year, the Project Management course is taught. All groups were managed in the same manner. We carried out the questionnaire after the teams were created.

### 2.2. Procedure

A cross-sectional design was developed. The questionnaire was distributed using Google Forms during the first week of the corresponding academic year. The answers were anonymous, voluntary, and self-administered. Only completed questionnaires were considered. The questionnaire was divided into two blocks: the first one includes descriptive academic variable (course level and topic), whereas the second block corresponds to the Eysenck Personality Questionnaire Revised-Abbreviated (Francis et al., 1992) and The Riso–Hudson Enneagram Type Indicator (Riso & Hudson, 1999a). The instruments of the second block were then used to classify the participants into their corresponding enneatypes. Although the data are anonymized, the project was authorized by the ethics committee of the institution.

During class time, students developed a product in several teams using Agile methodologies, in particular one of their most important framework called Scrum (Takeuchi & Nonaka, 1986). Scrum is based on an incremental working context, mainly used in the software development industry. The principles of Scrum are described in the Manifesto for Agile Software Development (Beck et al., 2001), as Scrum (Scrum.org, 2020) is one of these agile frameworks and related in (Capelli, 2018).

## 3. Instruments

The EPQR-A questionnaire (Eysenck Personality Questionnaire Revised-Abbreviated) (Francis et al., 1992) was originally introduced by Eysenck (1970) and Eysenck and Eysenck (1975). The instrument is made up of 24 items divided into four summative subscales (neuroticism, extraversion, psychoticism, and lie scale), each one composed of 6 items. The answer for each item is dichotomous, where 1 = yes and 0 = no. Therefore, the scores for each subscale range from 0 to 6. Several studies have validated the Spanish version of the questionnaire (Sandín et al., 2002; González et al., 2021) with optimal psychometric properties. In this sense, the Kuder–Richardson 20 internal consistency index for (González et al., 2021) was 0.824 for extraversion, 0.713 for neuroticism, 0.567 for lie scale, and 0.434 for psychoticism. Similar internal consistency results were found by (Sandín et al., 2002).

The Riso–Hudson Enneagram Type Indicator, RHETI (Riso & Hudson, 1999a), is a test used to identify complete personality types from nine basic profiles, the so-called enneatypes. This provides an overview that identifies the relative strengths and weaknesses of the nine enneatypes within their overall personality. The instrument has two versions, the first one is made up of 144 paired statements with a forced-choice answer scale, whereas the second version is an abbreviated test composed of two three-choice questions. The psychometric properties have been validated by several authors on different samples (Newgent et al., 2004; Giordano, 2008). The overall internal consistency of the instrument was 72%, which is above the minimum acceptable cut-off of 70%. Specifically, for various personality types, internal consistency ranges from 56% to 82% accuracy.

In this study, we used a license-free version of the abbreviated test, which, as already mentioned, is made up of two questions (Scott, 2011). Each question consists of three options, each reflecting the general attitudes and behaviors with which the respondent should identify. Only one option can be chosen for each question. So, each pair of chosen answers corresponds to one of the following nine types of personalities (Sutton et al., 2013).

1. Reformer: Characterized by being ethical and conscientious, possessing a strong sense of right and wrong.
2. Helper: Known for being sympathetic, sincere, and kind.
3. Achiever: Characterized by their self-confidence, attractiveness, and charm.
4. Individualist: Characterized for being self-conscious, sensitive, reserved, and quiet.
5. Investigator: Sharp, perceptive, and curious. They are able to concentrate and focus attention on developing complex ideas and skills.
6. Loyalist: Trustworthy, hard-working, and responsible, but they can also be defensive, evasive, and high-strung; they work themselves into a state of stress while complaining about it.
7. Enthusiast: Versatile, optimistic, and spontaneous; playful, lively, and practical; they can also be over-embracing, disorganized, and undisciplined.
8. Challenger: Self-confident, strong, and assertive. Protective, resourceful, and determined; they are also proud and domineering.
9. Peacemaker: Conformist, trusting, and stable. They are affable, kind, easily accommodating, and supportive, but may also be too willing to compromise with others to keep the peace.

There is a general lack of work applying the Enneagram in teams to determine personality, although some works validate its use in teams. For instance, Scott (2011) presents an analysis about each type of personality and the utility of the model. This fact has motivated us to opt for this model, in addition to the license-free nature of its use.

## 4. Data Analysis

The R statistical software and programming language version 4.1.0 (R Core Team, 2021) was used to apply Analysis of Variance (ANOVA). For the estimations of the response variable for all the possible combinations, the ‘emmeans’ R package was used (Lenth, 2022). This package computes the Estimated Marginal Means through the parameter estimates of the ANOVA model. Before that, normality and homoscedasticity were not rejected (*p* > 0.05). The ANOVA is a fundamental contrast in the analysis of experimental results, where the results of various ’treatments’ or ’factors’ are compared with respect to a dependent variable or variable of interest (Cano et al., 2012). The response variable is the final mark obtained by each group, on a 0–10 scale. Each group was directly assigned a single collective mark.

To test the hypothesis, we studied the effects of the enneatypes in the teams’ marks and how the different combinations may affect the performance. We used a two-way ANOVA. This statistical procedure allows us to control the values of independent variables and then measure the value of the response variable to discover the levels of the independent variables that improve the performance of the response. In this case, the experiment was not purely randomized, as students cannot be randomly assigned to a given personality (factors), but they already have an inherent one. Nonetheless, randomness in the teams’ composition is assured, as within each course students were assigned to teams using a simple random sampling. This process was carried out by the instructors. The randomization was consistently applied across all courses by each instructor, all of whom are experienced in agile frameworks and team-based teaching. Regarding the learners, they received a briefing of the methodology applied in the first course taught chronologically. Moreover, the course is considered a blocking factor. The experimental unit is the team, the response variable is the mark achieved by the team during the course, and the explanatory variables (factors) are the nine enneatypes (whether there is or not at least one member with the enneatype in the team).

The specific two-way ANOVA procedure used is the so-called two-level factorial design, with k = 9 factors: 1 per personality type. Each factor has two levels (yes = the personality type is present in the team; and no = the personality type is not present in the team). The two-level factorial design is particularly suited to mitigate the issues of small and uneven samples, as some enneatypes have very few or no participants in certain courses, which may lead to potential skewness in data interpretation (Montgomery, 2017; Kirk, 2013). With this procedure, we can determine whether the combination of certain enneatypes in a team will significantly increase or decrease the final mark, that is, allowing us to confirm or reject Hypotheses 1 and 2.

## 5. Results

### 5.1. Descriptive Statistics

Table 1 shows the frequencies of participants with each enneatype in the different topics: Software Processes (SPs), Software Architecture Design (SAD), Software Quality (SQ), and Project Management (PM), as well as the total count for each topic. In SP, the enneatype that has turned out to be most common is the helper, with 18 persons, and the least common is the researcher, with no answers. In SAD, the most common enneatype is also number 2, and the one with the least number of participants is type 5. In SQ, the helper is also the most common among the participants, and the challenger the least common. Finally, in PM, the most common enneatype is the helper, and the least is the achiever.

Table 2 shows the mean and standard deviation of the response variable for each enneatype and topic. Empty cells correspond to enneatypes not represented in the corresponding topic.

Table 3 shows the Pearson correlations between the enneatypes. Only the helper enneatype has significant correlations with other enneatypes, although all of these correlations have low magnitudes, with absolute values less than 0.03.

Table 4 shows descriptive statistics of the marks for each topic. It includes mean, standard deviation, range, minimum and maximum, percentiles, and variation coefficient.

### 5.2. One-Way Analysis of Variance

**Figure 1:**
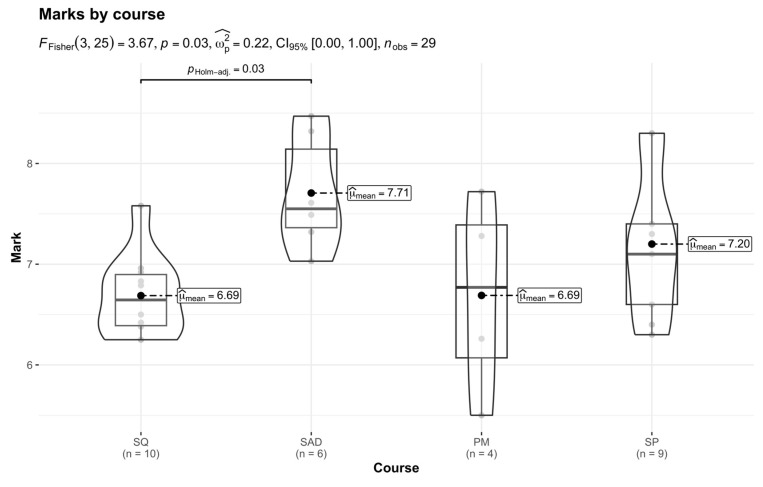
Comparison of marks between courses.

The results of the one-way analysis of variance showed significant differences between courses. The significant differences were between software architecture design (M = 7.71, SD = 0.57) and software quality (M = 6.69, SD = 0.41), with the *p*-value of the multiple comparison being 0.03 (*p* < 0.05). The plot in Figure 1 is a combination of boxplots and violin plots, and it includes a comparison of marks between courses. Individual marks are the semi-transparent points, and the means of each plot are remarked. The F test results are shown in the plot’s subtitle.

### 5.3. Two-Way Analysis of Variance

The complete two-way ANOVA model has a total of 45 terms: 9 main effects and (9 2) = 36 two-way interactions. However, as mentioned above, not all the combinations were met in the ANOVA, and therefore some interaction effects could not be estimated.

As a result of the stepwise selection, a reduced model with 25 terms is reached. Given that R^2^ = 0.78, the model explains 78% of the variability of the marks. Table 5 shows the estimates of the effects, as well as the *p*-values associated with their significance hypothesis test.

Regarding the main effects, teams with enneatype 3 (the achiever) members increase the average mark by 1.494 points, and those with enneatype 6 increase the average by 1.828 points. But if it contains both at the same time, the average mark drops by 3.522 points. Similarly, the combination 2–7 increases the mean mark by 2.604 points (its main effects being non-significant) and the combination 1–4 is the worst of all (lowering the mark by 3.927 points). These effects are understood as ceteris paribus, i.e., with all other factors remaining the same. The interaction plots below clearly show these effects. These results support H_1_.

**Figure 2:**
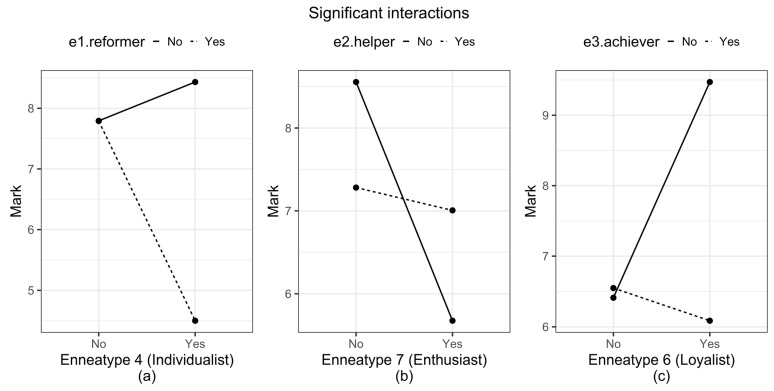
Most significant interactions between enneatypes. (**a**) Interaction between enneatypes 1 (the reformer) and 4 (the individualist); (**b**) Interaction between enneatypes 2 (the helper) and 7 (the enthusiast); (**c**) interaction between enneatypes 3 (the achiever) and 6 (the loyal).

Graphically, Figure 2 shows the most significant interactions. In Figure 2a, we can see the interaction between enneatypes 1 (the reformer) and 4 (the individualist). When they interact, the marks decrease by 3.927 points on average. These results support H_2_. Figure 2b shows the interaction between enneatypes 2 (the helper) and 7 (the enthusiast). We can see that if a team does not have type 7, the average mark is higher than if it also does not have type 2. But if the team has both, the grade is higher. In Figure 2c, the interaction between enneatypes 3 (the achiever) and 6 (the loyal) is shown. This combination does not appear in the model defined in theory but is relevant in the results. When it contains both, the average mark drops by 3.522 points.

After the main effects and two-way interactions analysis, we computed the estimated marginal means of the combinations for the enneatypes in any significant term, i.e., all but investigator, challenger, and pacemaker. This resulted in 26 = 64 possible combinations. Table 6 shows the top 5 best team compositions. The best combination for a team is reformer, achiever, individualist, loyalist, and enthusiast, without helper.

Table 7 shows the top five worst team compositions. Reformer combined with achiever, individualist, and enthusiast is the worst combination in a team, which is consistent with the interaction analysis.

In summary, loyalist is a key enneatype: when it is in the team, the average marks are better, and when it is not, the average marks are worse.

## 6. Discussion

### 6.1. Practical Implications

From a practical perspective, this study offers guidance for team formation in organizational settings. By considering the compatibility of personality types, specifically enneatypes, team leaders and managers can strategically form teams that are more likely to perform well. The results indicate that the presence of the loyalist enneatype can significantly improve team outcomes due to its characteristics of trustworthiness, responsibility, and adaptability (Guzzo & Shea, 1992; Snow & Walton, 2018). Conversely, combinations of less compatible types, such as the reformer and individualist, may negatively impact team performance, suggesting that managers should be cautious when pairing these personality profiles. Adding a loyalist to the team may help mitigate potential conflicts or inefficiencies (Appelo, 2010). Additionally, the complementary nature of the enthusiast and helper enneatypes suggests that fostering teams with these personality profiles could boost morale and productivity (Conard, 2006).

The results of this study can be explained by the inherent characteristics of the Enneagram types and their influence on group dynamics. For example, the loyalist’s responsibility and reliability foster a more stable and cooperative work environment, which can enhance overall performance. In contrast, less compatible types, such as the reformer and individualist, may cause conflicts due to differing approaches to decision-making and teamwork. As research in this area continues, more empirical relationships may emerge, offering a deeper understanding of how personality profiles interact within teams. These insights could help predict team behaviors and outcomes, reinforcing the applicability of these theories in future team formations.

In conclusion, the research provides a practical tool for team formation, suggesting that understanding and leveraging personality traits can lead to more effective and harmonious teams.

### 6.2. Theoretical Implications

This study contributes to the theoretical understanding of how personality types, specifically enneatypes, influence team performance. The findings suggest that certain combinations of personality types can either enhance or impair team outcomes, shedding light on the complex interplay between individual traits and collective success. For instance, the loyalist’s presence within teams is associated with improved performance, which aligns with previous research on trust and responsibility as key factors in effective team dynamics (Kozlowski & Ilgen, 2006). Additionally, the positive interaction between the enthusiast and helper enneatypes emphasizes the significance of personality compatibility in fostering optimal team collaboration (Conard, 2006).

These results offer a complementary perspective on team composition theories, confirming that personality-based team formation could provide helpful insights for refining models of team effectiveness and group dynamics. Future research will provide empirical data to validate or adjust these relationships, encouraging the emergence of new theories on team dynamics.

## 7. Limitations

This study has the following limitations. Firstly, concerning the threats to the validity of this work, the experiment was not purely randomized, as students cannot be randomly assigned to a given personality trait. Therefore, we have not covered all the possible combinations of Enneagrams in a team, and, as a consequence, we have an unbalanced design. This is a realistic situation, as, traditionally, members are randomly assigned to groups. A comprehensive study incorporating all conceivable combinations of personality types would offer more definitive findings regarding certain combinations that were not assessable due to the inherent randomness of this study. Nevertheless, the results of our study remain consistent with the personality model used, and, hence, the ability for generalization cannot be disregarded. In this sense, this study could be replicated using other personality models, such as the EPQR by Eysenck and Eysenck (1975); secondly, the small sample sizes within the combinations and the possible lack of heteroscedasticity may affect the power of the statistical tests; lastly, when people fill out a personality test, there is a tendency to endorse general descriptions of their personality as an accurate reflection of themselves. This phenomenon, known as the Forer effect (Forer, 1956), could lead to inaccurate results in some personality tests. This problem could be ameliorated by using additional personality tests or by conducting personal interviews; thirdly, we used an abbreviated instrument, although more psychometrically sound measures of the Big Five are available (Mammadov, 2022; Gosling et al., 2003). Moreover, we have assumed the theoretical factor structure of the instrument. In fact, an exploratory factor analysis may be reserved for the development of a new instrument entirely. The aim of this study was not to identify the underlying variables or factors of the instrument, but rather to detect the importance of the existing ones (Enneagrams) and their interactions. However, this could lead to a lack of consistency that should be taken into account for future studies. In this regard, we plan to analyze the psychometric properties of this instrument using exploratory and confirmatory factor analysis; and finally, it would be interesting to explore how interactions between similar Enneagram types, such as having two or more repeated types within the same team, impact team dynamics and project outcomes. Moreover, key issues, such as potential cultural biases, the impact of team dynamics beyond personality types, the limited scope of the study, the interaction between certain enneatypes in terms of underlying psychological mechanisms, and considering alternative statistical methods are limitations to be considered for future studies.

## 8. Conclusions

In conclusion, the Enneagram framework can be used as a preliminary instrument for identifying potential team members for future projects. By doing so, teams can be strategically assembled based on the most effective combinations of personality types, thereby enhancing overall team efficiency. This approach not only acknowledges the importance of individual differences but also maximizes the collective strengths of team members, ultimately leading to more successful project outcomes.

## Figures and Tables

**Table 1:** Participants frequency for each enneatype and course (only valid responses considered).

Class	SPs	SAD	SQ	PM	Total
Reformer	9	6	10	1	26
Helper	18	10	15	7	50
Achiever	5	4	7	0	16
Individualist	4	2	5	2	13
Investigator	0	0	5	3	8
Loyalist	3	4	5	3	15
Enthusiast	6	3	6	1	16
Challenger	6	2	2	1	11
Peacemaker	2	1	5	1	9
Total	53	32	60	19	164

SPs = Software Processes; SAD = Software Architecture Design; SQ = Software Quality; and PM = Project Management.

**Table 2:** Mean (standard deviation) of the response variable for each enneatype and topic.

Class	SPs	SAD	SQ	PM	Total
Reformer	6.96 (0.63)	7.95 (0.47)	6.63 (0.25)	6.26 (0)	6.95 (0.72)
Helper	7.09 (0.68)	7.73 (0.47)	6.39 (0.23)	6.39 (1.11)	6.9 (0.64)
Achiever	7.36 (0.62)	7.29 (0.19)	6.71 (0.44)	- (-)	7.12 (0.36)
Individualist	6.85 (0.52)	7.03 (0)	6.89 (0.09)	6.55 (0.65)	6.82 (0.20)
Investigator	- (-)	- (-)	6.61 (0.33)	6.87 (1.02)	6.74 (0.18)
Loyalist	6.97 (0.51)	7.42 (0.57)	6.81 (0.19)	7.09 (0.75)	7.07 (0.26)
Enthusiast	7.52 (0.71)	8.39 (0.11)	6.63 (0.50)	6.26 (0)	7.02 (0.95)
Challenger	7.5 (0.62)	8.32 (0)	7.20 (0.53)	6.26 (0)	7.32 (0.85)
Peacemaker	7.3 (1.41)	7.32 (0)	6.55 (0.34)	7.07 (0)	7.06 (0.44)

SPs = Software Processes; SAD = Software Architecture Design; SQ = Software Quality; and PM = Project Management.

**Table 3:** Pearson correlations between each pair of the constructs (enneatypes).

	1	2	3	4	5	6	7	8
1. Reformer	-							
2. Helper	−0.28 **	-						
3. Achiever	−0.14	−0.21 **	-					
4. Individualist	−0.13	−0.19 *	−0.10	-				
5. Investigator	−0.10	−0.15	−0.07	−0.07	-			
6. Loyalist	−0.14	−0.21 **	−0.10	−0.09	−0.07	-		
7. Enthusiast	−0.14	−0.21 **	−0.11	−0.10	−0.07	−0.10	-	
8. Challenger	−0.12	−0.17 *	−0.09	−0.08	−0.06	−0.08	−0.09	-
9. Peacemaker	−0.10	−0.16 *	−0.08	−0.07	−0.05	−0.08	−0.08	−0.06

* *p* < 0.05, ** *p* < 0.01.

**Table 4:** Descriptive statistics of the marks for each course in the study.

	SPs	SAD	SQ	PM
Mean	7.20	7.71	6.69	6.25
Std. Dev	0.73	0.57	0.41	1.00
Min	6.30	7.03	6.25	5.50
Q1	6.60	7.32	6.38	5.88
Median	7.10	7.55	6.64	6.77
Q3	7.40	8.32	6.92	7.50
Max	8.30	8.47	7.58	7.72
CV	0.10	0.07	0.06	0.15
N	53	32	60	19

SPs = Software Processes; SAD = Software Architecture Design; SQ = Software Quality; and PM = Project Management.

**Table 5:** ANOVA model estimates of the effects after stepwise selection.

Term	Estimate	*p*-Value
Intercept	7.764	0.020 *
Software Architecture Design	−0.567	0.516
Project Management	−0.752	0.181
Software Processes	1.230	0.012 *
Reformer	−1.179	0.533
Helper	−1.177	0.461
Achiever	1.494	0.047 *
Individualist	0.034	0.966
Investigator	−0.359	0.689
Loyalist	1.828	0.017 *
Enthusiast	−3.131	0.069
Challenger	2.827	0.053
Peacemaker	−0.215	0.053
Reformer × helper	−1.502	0.269
Reformer × individualist	−3.927	0.020 *
Reformer × investigator	0.815	0.317
Reformer × loyalist	2.465	0.101
Reformer × enthusiast	1.344	0.186
Reformer × challenger	−0.772	0.459
Helper × enthusiast	2.604	0.031 *
Helper × peacemaker	1.307	0.164
Achiever × investigator	−1.872	0.088
Achiever × loyalist	−3.522	0.028 *
Achiever × investigator	−1.872	0.088
Achiever × enthusiast	−0.841	0.202
Individualist × investigator	1.211	0.151

* *p* < 0.05.

**Table 6:** Top 5 best estimated marginal means (EMMs) by team compositions.

Reformer	Helper	Achiever	Individualist	Loyalist	Enthusiast	EMM
Yes	No	Yes	Yes	Yes	Yes	12.00
No	No	No	Yes	Yes	No	11.34
No	Yes	No	Yes	Yes	No	10.81
Yes	Yes	No	No	Yes	Yes	10.79
No	No	No	No	Yes	No	10.70

**Table 7:** Top 5 worst estimated marginal means (EMMs) by team compositions.

Reformer	Helper	Achiever	Individualist	Loyalist	Enthusiast	EMM
Yes	No	Yes	Yes	No	Yes	2.35
Yes	Yes	No	Yes	No	No	2.40
Yes	No	No	Yes	No	Yes	2.64
Yes	Yes	Yes	Yes	No	Yes	2.93
Yes	Yes	Yes	Yes	No	No	2.95

## Data Availability

Data is unavailable due to privacy or ethical restrictions.
